# Adding Zooplankton to the OSMAC Toolkit: Effect of Grazing Stress on the Metabolic Profile and Bioactivity of a Diatom

**DOI:** 10.3390/md19020087

**Published:** 2021-02-03

**Authors:** Renate Døving Osvik, Richard Andre Ingebrigtsen, Maria Fredrika Norrbin, Jeanette Hammer Andersen, Hans Christian Eilertsen, Espen Holst Hansen

**Affiliations:** 1 Marbio, Norwegian College of Fishery Science, Faculty of Biosciences, Fisheries and Economics, UiT–The Arctic University of Norway, N-9037 Tromsø, Norway; jeanette.h.andersen@uit.no (J.H.A.); espen.hansen@uit.no (E.H.H.); 2 Microalgae and Microbiomes, Norwegian College of Fishery Science, Faculty of Biosciences, Fisheries and Economics, UiT–The Arctic University of Norway, N-9037 Tromsø, Norway; richard.a.ingebrigtsen@uit.no (R.A.I.); hei000@post.uit.no (H.C.E.); 3 Arctic Marine System Ecology, Department of Arctic and Marine Biology, Faculty of Biosciences, Fisheries and Economics, UiT–The Arctic University of Norway, N-9037 Tromsø, Norway; fredrika.norrbin@uit.no

**Keywords:** diatoms, microalgae, biotechnology, biodiscovery, OSMAC, cultivation

## Abstract

“One strain many compounds” (OSMAC) based approaches have been widely used in the search for bioactive compounds. Introducing stress factors like nutrient limitation, UV-light or cocultivation with competing organisms has successfully been used in prokaryote cultivation. It is known that diatom physiology is affected by changed cultivation conditions such as temperature, nutrient concentration and light conditions. Cocultivation, though, is less explored. Hence, we wanted to investigate whether grazing pressure can affect the metabolome of the marine diatom *Porosira glacialis*, and if the stress reaction could be detected as changes in bioactivity. *P. glacialis* cultures were mass cultivated in large volume bioreactor (6000 L), first as a monoculture and then as a coculture with live zooplankton. Extracts of the diatom biomass were screened in a selection of bioactivity assays: inhibition of biofilm formation, antibacterial and cell viability assay on human cells. Bioactivity was found in all bioassays performed. The viability assay towards normal lung fibroblasts revealed that *P. glacialis* had higher bioactivity when cocultivated with zooplankton than in monoculture. Cocultivation with diatoms had no noticeable effect on the activity against biofilm formation or bacterial growth. The metabolic profiles were analyzed showing the differences in diatom metabolomes between the two culture conditions. The experiment demonstrates that grazing stress affects the biochemistry of *P. glacialis* and thus represents a potential tool in the OSMAC toolkit.

## 1. Introduction

The number of investigations of bioactive compounds from marine microorganisms has increased over the last few decades [1]. This is a natural development, since access and expeditions to the various parts of the water that covers 70% of the earth’s surface have increased during the recent years. These diverse aquatic biotopes are inhabited by millions of species of microorganisms, both prokaryotic and eukaryotic. Using high throughput DNA sequencing, it has been found that there are a number of biosynthetic gene clusters (BGCs) that are linked to the production of secondary metabolites. These are not necessarily expressed when the microorganisms are cultured under laboratory conditions [2]. This realization eventually led to a concept termed “one strain many compounds” (OSMAC) [3], stating that many microorganisms have the potential to produce a broad range of secondary metabolites, but that only a few are synthesized under specific growth conditions. It is therefore possible to alter growth conditions such as nutrient concentration, physical conditions, trace elements and the presence of other species through cocultivation to induce the production of a wider range of secondary metabolites. The OSMAC approach has led to the discovery of novel bioactive compounds. Uchoa et al. found that a strain of *Aspergillus niger* produced a novel furan ester derivative with bioactivity against a colon carcinoma cell line (HCT-116), when cultivated in MPDB (malt peptide dextrose broth medium) [4]. Cocultivation of two *Aspergillus* sp. strains and the marine fungi *Avicennia marina* led to the isolation of a new alkaloid with antibacterial activity against *E. coli* [5]. These examples and several others show us that OSMAC has become an important tool in the field of natural product biodiscovery [2,5,6,7,8,9].

One of the most diverse groups of marine microorganisms are the diatoms [10,11], which are a largely unexplored source of chemical diversity. Most OSMAC cultivation experiments have involved prokaryotes, and there are only a few studies involving marine microalgae. A study by Lauritano et al. [12] showed that nitrogen concentration influenced anticancer and antibacterial properties of the diatom *Skeletonema marinoi*. A study by Ingebrigtsen et al. [13] demonstrated that the bioactivity of five diatom species changed with different light and temperature regimes, e.g., *S. marinoi* only showed anticancer activity when cultivated at high temperature. Studies like these show that the OSMAC approach can be used on diatoms as well as bacteria. 

Changing cultivation conditions for microorganisms is a way of mimicking natural cultivation conditions in the laboratory. In nature, microorganisms like diatoms coexist with a plethora of other organisms. There is a constant competition between species competing for the same recourses, as well as pressure from predators. The concentration of diatoms in the ocean fluctuates throughout the year. During the annual spring bloom in Northern Temperate and Arctic waters the diatoms dominate the phytoplankton community, reaching concentrations up to 6–10 × 10^6^ cells L^−1^ [14]. The diatom bloom is followed by an increase in the number of grazing mesozooplankton such as copepod species [15,16], and microzooplankton such as heterotrophic protozoans, dinoflagellates and flagellates. Diatoms have for many decades been regarded as the main food source for many zooplankton species and are highly important for the transfer of nutrients up the food chain [17,18]. This relationship has been challenged after several studies found possible toxic effects on copepods feeding on diatoms [19,20,21,22,23,24,25,26,27,28]. These studies have investigated the effect of diatom species such as *Phaeodactylun tricornutum, Pseudo-nitzschia delicatissima, Thalassiosira gravia and Thalassiosira rotula,* and about 10 other species. Studies have revealed reduced hatching success in copepods fed a diet of *T. rotula*, and during a bloom of *Skeletonema costatum* and *P. delicatissima* [29,30]. Chaudron et al. [31] found that an increasing proportion of the diatoms *P. tricornutum* and *T. rotula* in the diet led to a reduction in hatching success. Diatoms in the diet can have an insidious effect, i.e., affecting the offspring rather than the consumer itself, and Poulet et al. [32] found teratogenic effects on nauplii larvae leading to fatal abnormalities. The compounds that have been deemed culprits in many of these studies are oxylipins, especially polyunsaturated aldehydes (PUAs) [30]. PUAs are not synthesized inside the cells, but are rather produced enzymatically by lipoxygenase/hydroperoxide lyase from polyunsaturated long chained fatty acids seconds after the rupture of cell membranes due to feeding [27,33]. On the other hand, studies have shown that copepods release chemical signals and cues, often polar lipids called copepodamides, that can trigger a response in microalgae [34,35]. Copepodamides have been shown to affect the morphological features of *Skeletonema marinoi,* such as a decrease in chain lengths [36,37]. Other reported effects on diatoms have been an induced production of the toxin domoic acid by *Pseudo-nitzschia* [38].

Based on the aforementioned studies it is therefore reasonable to think that diatoms can be affected by stress and thus alter their biochemistry, and we hypothesized that diatoms could change their metabolite expression in response to grazing pressure. Metabolomics can be used to investigate the diatoms at a specific time under specific conditions, e.g., when exposed to grazing pressure, in order to reveal changes in the metabolome [39,40]. Further, we hypothesized that such changes in metabolic expression could be detected as a change or an increase in bioactivity in cell-based bioassays; bacterial growth inhibition assays, the inhibition of biofilm formation and the viability of human cell lines, and that grazing pressure could trigger the production of compounds leading to higher bioactivity. Inducing stress in diatom cultures by cocultivation with zooplankton could thus be a potential new “tool” in the OSMAC toolkit, i.e., a method to discover new bioactive metabolites.

In this study we cultivated the marine temperate diatom *Porosira glacialis* (Grunow) Jörgensen 1905 in a large photobioreactor (PBR), first as a monoculture and then in coculture with zooplankton from a nearby costal bay. More specifically the aim of the study was to investigate whether the presence of a small but varied grazer population could alter the *P. glacialis*’ biochemistry and bioactivity.

## 2. Results

### 2.1. Cultivation of P. glacialis and Cocultivation with Zooplankton

**Figure 1:**
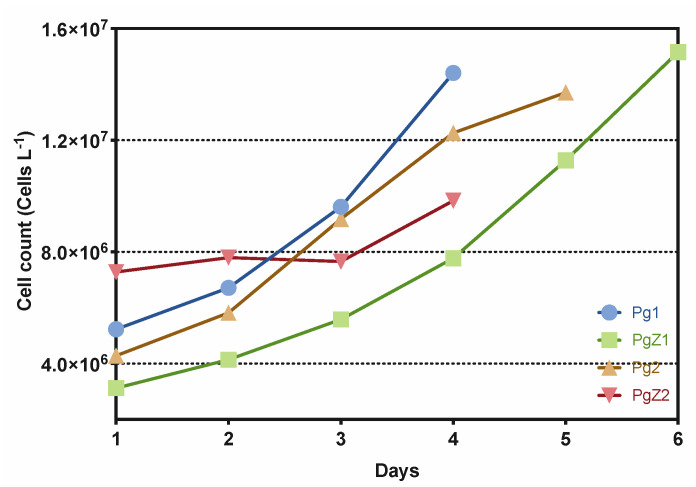
Cell count of *P. glacialis* in cells L-1 vs. time (days) for all four cultures (monocultures Pg1 and Pg2, and cocultures PgZ1 and PgZ2).

Four nonaxenic batch cultures were cultivated during the experiment: two monocultures abbreviated Pg1 and Pg2 and the two coculture samples abbreviated PgZ1 and PgZ2. All were closely monitored to keep track of whether the cells were healthy and that we had no contamination of unwanted organisms in the culture tanks. All cultures were in good growth condition throughout the cultivation period (Figure 1). Before harvest of the coculture, 10 × 1 L of the culture was transferred to clear flasks to enumerate the number of zooplankters per liter of culture. The concentrations of animals were 1.5 individuals L^−1^ for PgZ1 and 1.0 L^−1^ for PgZ2. The growth data of diatoms based on cell counts (Figure 1) showed that all cultures, except for PgZ2 (growth rate 0.1 doubling day^−1^), had a steady increase in cell numbers (growth rates 0.29–0.33 doublings day^−1^), and were all harvested during the exponential growth phase when the cell numbers had reached 12–15,000,000 cells L^−1^. The second coculture, PgZ2, obviously had a lag phase, but had started the exponential phase when it was harvested after four days. Harvest at a lower cell count was done due to time limitations on the experiment. See Supplementary Material for data on growth rates, temperature, pH, and nutrient concentrations (Figures S2–S6).

**Figure 2:**
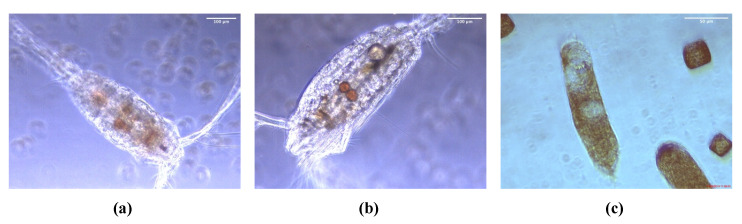
Micrographs (**a**) and (**b**) showing two *Oithona* sp. specimens which have *P. glacialis* biomass in the stomach. Micrograph (**c**) shows fecal pellets containing *P. glacialis*.

Taxonomic analysis of zooplankton species was done based on morphological traits. Both zooplankton batches were dominated by two genera: the cyclopoid copepod *Oithona* sp. and the calanoid copepod *Pseudocalanus* sp. (Table 1). PgZ1 had a higher diversity than PgZ2, and more species could therefore be identified. A few specimens of *Oithona* sp. from the cocultures were collected and investigated using a microscope to see whether they had fed on *P. glacialis* during the cultivation period. The micrographs in Figure 2 shows *P. glacialis* in the stomach (a and b), as well as in the fecal pellets (c) of *Oithona* sp.

### 2.2. Extraction and Fractionation

From the four cultures (Pg1, Pg2, PgZ1 and PgZ2) organic and aqueous extracts were made. As the crude extracts are complex chemical mixtures, they were separated using flash-chromatography. Each of the 4 samples was fractionated into 8 flash-fractions giving 64 fractions in total. All fractions were tested in all bioassays.

### 2.3. Inhibition of Biofilm Formation

**Figure 3:**
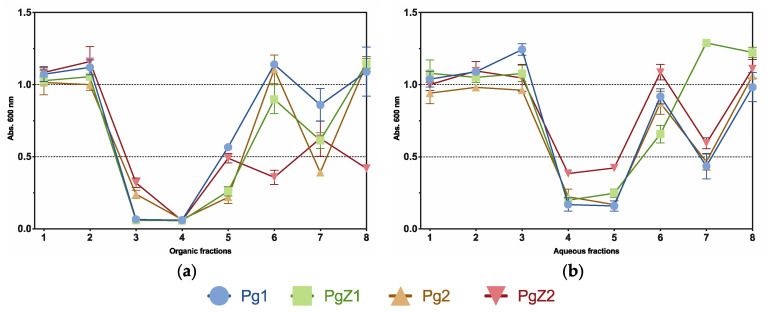
Activity profiles of all samples from biofilm inhibition assay, organic flash fractions from all four samples are shown in (**a**), while (**b**) shows the aqueous flash-fractions. All tested at 50 µg mL^−1^. All fractions with absorption readings below 0.25 at 600 nm are regarded as active.

All flash fractions (organic and aqueous) were tested for inhibition of biofilm formation and Figure 3 shows the bioactivity profiles of all flash fractions tested in the biofilm assay. The initial screening was performed using a concentration of 50 μg mL^−1^ and revealed 13 active fractions from a total of 64 tested. Out of the total number of 13 active fractions (absorption below 0.25 at 600 mn), 9 were organic and 4 from aqueous extracts. The activity profiles revealed a similar pattern for all fractions and samples tested, where the most activity was found in fractions 3, 4 and 5 for the organic samples (Figure 3a), and in fractions 4 and 5 for the aqueous samples (Figure 3b). Fraction 5 was the only active fraction from both organic and aqueous extracts. Based on the similarity in the activity profile of all samples (both aqueous and organic), there was no clear difference between the activity in fractions from monocultures or cocultures. These results show that grazing stress did not affect the activity on inhibition of biofilm formation.

### 2.4. Cell Viability

**Figure 4:**
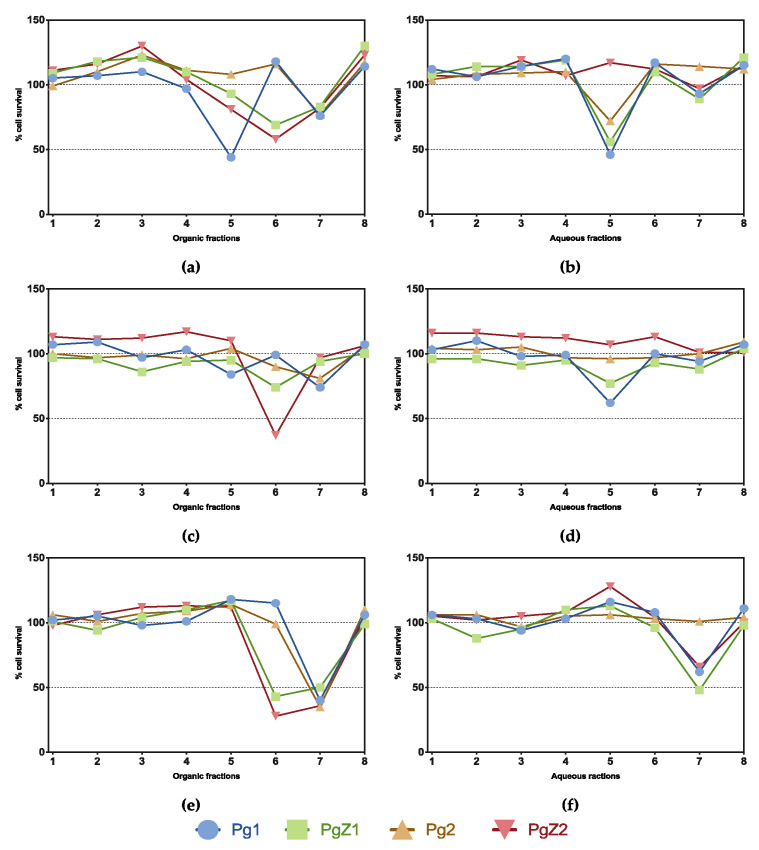
Overview of activity of all samples in cell viability assay against human colon carcinoma (HT29) (**a**,**b**), human melanoma (A2058) (**c**,**d**) and normal lung fibroblasts (MRC5) (**e**,**f**) at 50 μg mL^−1^. Fractions were regarded as active when cell viability was measured below 50%.

All samples, organic and aqueous, were tested for activity against the three human cell lines: HT29 human colon carcinoma, A2058 human melanoma, and MRC5 normal lung fibroblasts in a cell viability assay at 50 μg mL^−1^ (Figure 4). As the figure shows, activity was found against all three cell lines. Towards the HT29 colon carcinoma cells, the activity profile of the fractions from the different cultures were similar; the only difference was observed for Pg1, where both organic and aqueous fraction 5 were active (Figure 4a,b). Towards the melanoma cell line A2058, only organic PgZ2 fraction 6 showed activity (Figure 4c). No difference in the activity between the two cultivation conditions for the other fractions was detected. The bioactivity profile against the normal cell line MRC5 revealed a difference between monoculture and coculture samples; organic fraction 6 of the PgZ1 and 2 were active against the cells, while Pg1 and 2 were not (Figure 4e). This fraction also showed activity against the A2058 for one of the cocultures (PgZ2) as described above. The bioactivity profile against HT29 shows that organic fraction 6 of PgZ1 and 2 also had some effect on human colon carcinoma compared to Pg1 and 2.

### 2.5. Bacterial Growth Inhibition Assay

**Figure 5:**
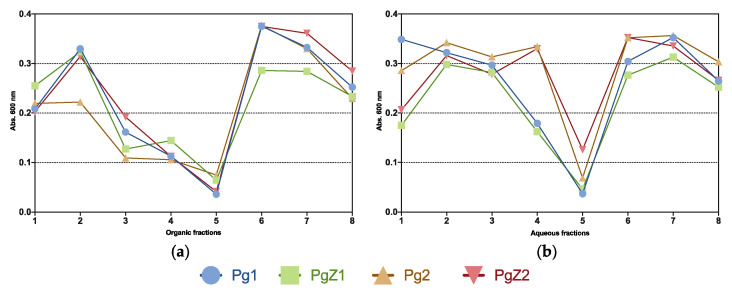
Overview of activity of all four samples against *Streptococcus agalactiae*. Organic fractions in (**a**) and aqueous fractions in (**b**). All tested at 50 μg mL^−1^. Fractions regarded as active with absorbance readings below 0.05 at 600 nm.

All samples were tested in a growth inhibition assay against the five bacterial strains *Staphylococcus aureus* (ATCC 25923), *Escherichia coli* (ATCC 25922), *Pseudomonas aeruginosa* (ATCC 27853), *Escherichia faecalis* (ATCC 29212) and *Streptococcus agalactiae* (ATCC 12386) at 50 μg mL^−1^. The results in Figure 5 showed that fraction 5 (both organic and aqueous) from all four samples was active against one of the strains, *Streptococcus agalactiae*. There was no activity against the other strains, and no difference in activity of the two cultivation treatments. This showed that grazing pressure had no obvious effect on antibacterial activity of *P. glacialis*.

### 2.6. Metabolic Profile

**Figure 6:**
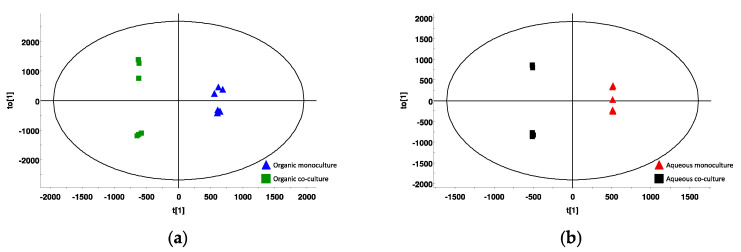
Scores plot of metabolic profiles. Plot (**a**) is based on collected markers from organic samples from monocultures and cocultures, and (**b**) are based on collected markers from aqueous extracts of monocultures and cocultures.

Crude extracts of all samples (organic and aqueous) were analyzed using UHPLC and HR-MS to obtain metabolic profiles of the extracts. The UHPLC-HR-MS chromatograms of the extracts were very complex, and in order to identify differences between the different samples, we analyzed the data in a metabolomics workflow. The scores’ plots (Figure 6) are based on the collected markers (i.e., combinations of a mass and a retention time) from all samples. The monoculture and coculture samples were well separated both for the organic samples (Figure 6a) and the aqueous extracts (Figure 6b), showing that there are variations in the metabolic profiles of the two cultivation conditions. All monoculture samples clustered together, meaning that there was little variation within the group. The two different coculture samples (i.e., PgZ1 and PgZ2) grouped separately for both the organic and aqueous extracts, indicating that there were some differences in metabolic profiles between the two cultivations. However, as we did not observe any significant differences in bioactivity between these two cocultivations, we assume that the difference is due to variations in cultivation temperature, as sample PgZ2 was cultivated at lower average temperature than the others.

**Figure 7:**
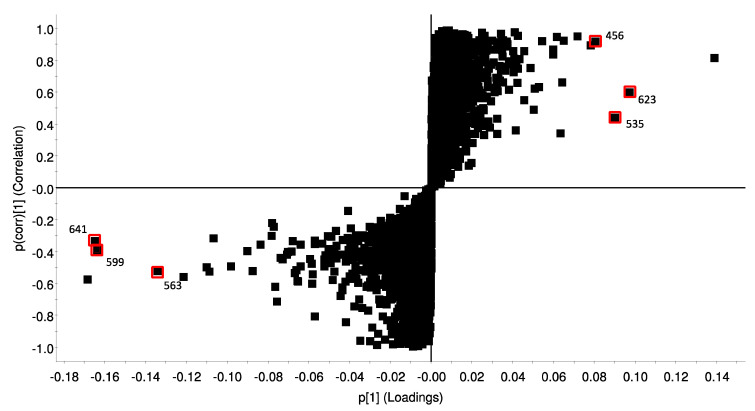
S-plot of organic samples of cocultures: PgZ1 and 2 (=−1, bottom left) and monocultures: Pg1 and 2 (= 1, top right) The x axis denotes the contribution of the markers to the differences, and the y-axis shows the confidence in the contribution. Markers highlighted in red are those where a matching compound was identified.

To reveal differences between the metabolic profiles, the organic extracts of both cocultures (PgZ1 and 2) and monocultures (Pg1 and 2) were compared in a s-plot (Figure 7). The x-axis of the plot denotes the contribution of the marker to the differences between the samples, and the y-axis denotes the confidence in the contribution. The markers in the left lower corner were characteristic for the coculture samples, while the markers in the upper right corner were characteristic for the monoculture samples. The 5 markers from each sample set contributing the most to the differences were attempted identified by using the MS analysis software. The markers where a matching compound was identified by the Dictionary of Marine Natural Products or MarinLit are marked in red in Figure 7. Elemental composition calculations of the markers were done using HR-MS data in ES+.

The two markers in the coculture samples *m/z* 641.4193 and *m/z* 599.4088 were identified as the carotenoids halocynthiaxanthin acetate (C_42_H_56_O_5_) and halocynthiaxanthin (C_40_H_54_O_4_), respectively. Both compounds were previously isolated from marine sources [41]. A third carotenoid with *m/z* 563.388 was identified as clathriaxanthin (C_40_H_50_O_2_), which has been isolated from marine sponges [42,43]. Two of the markers in the monoculture samples were identified as chlorophyll derivatives. The marker *m/z* 623.2855 (C_36_H_38_N_4_O_6_) was suggested to be a pheophorbidelike structure and marker *m/z* 535.2706 (C_33_H_34_N_4_O_3_) as pyropheophorbide b. The compound with *m/z* 456.2789 and elemental composition C_27_H_37_NO_5_ corresponded with a compound earlier found in marine derived fungi [44].

The active fraction 6 of PgZ1 and PgZ2 were analyzed using UPLC-HR-MS to search for compounds responsible for the activity. The chromatograms of the active fractions were compared to the nonactive fraction 6 of Pg1 and Pg2 to search for possible differences. No apparent differences were found in the chromatograms with respect to the presence of the more prominent metabolites, but there were numerous cases where the signal strength of compounds varied between the active and the inactive fractions. The max fold change of the total number of compounds found in the metabolomics dataset (of all four samples) showed that 4972/14280 compounds had a noticeable upregulation (fold change cut off > 5). Due to the high number of compounds with differences in abundances, it was not possible to attribute the differences in bioactivity to any specific metabolite. However, the manual dereplication resulted in finding signals corresponding to degradation products of chlorophyll such as pheophorbide and membrane components such as phosphocholines among the prominent metabolites.

## 3. Discussion

The aim of the present study was to investigate if the presence of a small but varied grazer population could alter the *P. glacialis* biochemistry and bioactivity, i.e., to evaluate if zooplankton grazing could be applied as a tool to induce the production of bioactive compounds.

Extracts of biomass cultivated with and without the presence of zooplankton were tested in three different bioactivity assays: human cancer cell viability, bacterial growth inhibition and inhibition of bacterial biofilm formation. The screening of the flash-fractions was done at a concentration of 50 μg mL^−1^, allowing comparisons of activity between fractions and assays. Fractions that were active at 50 µg mL^−1^ were retested at different concentrations after serial dilutions to evaluate dose–response effects. Using the same test concentrations of all the fractions in the initial screening allowed us to assess possible differences between the different cultivation conditions. All samples showed activity in all performed assays, confirming the bioactivity previously found in *P. glacialis* by Ingebrigtsen et al. [13].

As diatoms are known to change their bioactivity and metabolic profile in response to changing cultivation conditions such as light, temperature and nutrients [12,13,45] we aimed to minimize variations in cultivation conditions in order to prevent cultures from being influenced by changes other than the presence of the grazing zooplankton. Our experiment was carried out in a 6000 L vertical column mesocosm bioreactor at a cultivation facility located outdoors. Being located outdoors, the temperature of the cultures was dependent on the air and water temperature of the season, but as the experiment was conducted during winter the temperature was relatively stable, i.e., average cultivation temperatures were 4.3–5.4 °C during the cultivation of cultures Pg1, PgZ1 and Pg2. Only one culture, PgZ2, had a lower average temperature (1.0 °C). Temperature change has been shown to influence the antioxidant activity of *P. glacialis* but did not have any effect on anticancer activity [13].

Several calanoid copepods are known to enter hibernation and nonfeeding (diapause) during winter when the concentrations of microalga in the water column are negligible [46]. As our experiment was performed during winter, it was uncertain how our added zooplankton sample would react to the transition from dark, clear water to a dense diatom culture with 24 h illumination. Our investigation of the copepods under the microscope showed that they were still alive at the end of each cultivation period. Visual inspection of all the investigated specimens also confirmed that they were feeding on the diatoms. Furthermore, the fecal pellets found in the same samples contained partly digested *P. glacialis*. Previous studies have revealed that the small copepod *Oithona* sp., one of the dominating species in the samples, remains active during winter, feeding on what they find [47,48,49]. We thus concluded that the zooplankton were actively grazing on the diatoms. Regarding the total effect of the presence of grazing zooplankton on *P. glacialis* we also need to consider the concentration of zooplankton in the culture. At the peak of zooplankton abundances during the spring bloom, the number of copepods can be > 100 L^−1^ [38,50]. Such high concentrations constitute a major grazing pressure on the diatoms and other microalgae. Our experiment was performed with a concentration of 1–1.5 specimens per liter of culture, which in comparison is very low, especially when the diatom concentrations are considered. However, copepods can scare many more prey than they consume [37,51]. In addition, it is important to consider that the zooplankton was added to a monoculture of *P. glacialis* which had been growing without competition from any other species of phytoplankton or zooplankton for a long period of time. Although our samples were sieved, a higher concentration of zooplankton could have resulted in the extraction of metabolites from zooplankton, and not *P. glacialis* as was our aim. As we managed to keep conditions similar between treatments, and at an early stage of the experiment obtained evidence that the zooplankton were grazing on *P. glacialis*, we expect that the differences we observed were due to the presence of zooplankton.

The results from the bioactivity testing against bacteria revealed no significant difference in the bioactivity of the two cultivation conditions (*p* > 0.05 for all samples, statistical analysis in Table S2). The inhibition of biofilm formation assay revealed 14 active fractions in total, 9 from monoculture samples vs. 4 from coculture samples. As the results of the assay were based on the amount of biofilm present after 24 h of exposure to the flash-fractions, it cannot be excluded that the activity was due to growth inhibition of the bacteria itself rather than inhibition of biofilm formation. This can only be ruled out by a growth inhibition assay, which should be performed if the results of the biofilm activity assay are to be studied further. There were more active fractions from the monoculture samples, but the activity profile of the tested fractions showed similar patterns in all samples tested. These similarities could indicate that the difference in activity was due the concentration of active component in the fractions, and not due to different components in the fractions from the two different cultivation treatments. Bioactivity against the formation of bacterial biofilm in diatom extracts has been investigated to a lesser extent, and there are few studies showing the biofilm activity of marine diatoms [12]. Such activity can be a strategy by the diatoms to prevent bacterial growth on the surface of the diatom, in its phycosphere or immediate surroundings, but the stress of grazing pressure has no apparent effect on *P. glacialis*’ ability to inhibit the formation of biofilm. Large scale microalgal cultures will always contain bacteria. Bacteria are in fact often beneficial in diatom cultivation, and necessary to provide certain compounds needed by the diatoms, such as vitamin B_12_, iron and other trace elements [52]. The production of antibacterial compounds by the diatoms might not be beneficial when the bacteria present are not parasitic or in any way harmful for the diatoms. The presence of grazers such as copepods, on the other hand, might induce stress reactions in the diatoms that could lead to the synthesis of secondary metabolites with activity that could also have antibacterial effects. Our experiment showed no significant difference in antibacterial effect between the two cultivation conditions, and the total number of active fractions in the growth inhibition assay was low (5/64).

All samples were screened in a viability test against two cancer cell lines, human colon carcinoma and human melanoma, and activity was found against both cell lines. Cytotoxic activity in extracts of marine diatoms is known from previous studies [12,13], but the compounds responsible in those studies has not been identified. Sansone et al. [53] found anticancer activity against A549 (ATCC CCL185) human lung adenocarcinoma and COLO 205 (ATCC CCL-222) colon adenocarcinoma when exposing the cell lines to the diatom-derived PUAs 2-*trans*, 4-*trans*-decadienal, 2-*trans*,4-*trans*-octadienal and 2-*trans*,4-*trans*-heptadienal, all of which had anticancer activity, and no activity against the normal lung/brunch epithelial BEAS-2B cell line. Although activity was observed against both cancer cell lines, the activity profile showed no significant difference between the cultivation treatments, indicating that cocultivation with zooplankton had no obvious effect on the anticancer activity of *P. glacialis*. The viability assay was also conducted against normal lung fibroblasts (MRC5). This viability assay revealed a difference between the two cultivation treatments. Organic fraction 6 of PgZ1 and 2 were active and led to a decrease in cell viability, while fraction 6 of Pg1 and 2 had no effect on the cells. The same fraction also showed activity against A2058 for PgZ2. This shows that the presence of zooplankton induced the production of compound(s) that are toxic against normal lung fibroblasts. In a study by Ingebrigtsen et al. [54] field samples of microalgae and zooplankton from a spring bloom in the Barents Sea were screened for bioactivity and compared to a monoculture of *P. glacialis*. In the study, field samples composed of only microalgae, only zooplankton and the two combined were all active against normal lung fibroblasts, while the cultivated monoculture of *P. glacialis* was not active in the same assay. These results compared to those from the current study might suggest that diatoms under stressful conditions produce compounds that are toxic to normal human cell lines. The active fractions were analyzed using UPLC-HR-MS to search for possible differences that could reveal which compounds were responsible for the activity in fraction 6 of PgZ1 and PgZ2. Dereplication of some prominent peaks of the chromatograms revealed signals corresponding to chlorophyll derivatives such as pheophorbide and membrane components such as phosphocholines. Pheophorbidelike compounds has previously been shown to have anticancer and cytotoxic properties [55]. In the metabolomic analysis of the extracts, several carotenoid compounds were found to contribute most to the differences in the cocultivated samples. Carotenoids such as fucoxanthin have shown anticancer activity in previous studies both in diatoms and green algae [56,57,58]. Analysis of the chromatograms revealed no apparent differences in metabolite composition, but rather a difference in the concentration of the metabolites, based on the signal strength of the peaks in the chromatogram. The fractions are highly chemically complex, and pin-pointing a culprit responsible for the specific cytotoxicity is difficult. However, based on evidence found in the LC-MS analysis of active fractions and metabolomics samples, the cytotoxicity might be attributed to chlorophyll degradation products or carotenoid compounds.

Grazing copepods are known to influence diatom morphology [36,59] and biochemistry [38]. Amato et al. [60] investigated the metabolomic and transcriptomic changes in *Skeletonema marinoi* after cocultivation with *Calanus finmarchicus* and *Centropages typicus* and found that there was an activation of stress response, and a change in lipid and nitrogen metabolism of the diatoms. Our analysis of the metabolomic data and metabolic profiles of the organic extracts show a difference between the samples from the monocultures and the cocultures, but this difference might not be detectable as a change in the bioactivity of the samples. Initial attempts to identify the compounds contributing the most differences were done. In the monoculture samples the markers were identified as a possible alkaloid and two pheophorbidelike compounds. Pheophorbide has been linked to grazing, as it is a pigment often found in fecal pellets of copepods due to the degradation of chlorophyll in the stomach [61]. In addition to phaeophytin a, pheophorbide is a known degradation product of chlorophyll a that we expected to be present in our samples [62]. Analysis of the stress response by Amato et al. [60] showed the downregulation of chlorophyll binding proteins when the diatom *Skeletonema marinoi* was exposed to copepod grazing. The compounds contributing most to the difference in the coculture sample set were identified as carotenoids. Carotenoids are one of the most abundant groups of pigments in nature, and play important roles in many physiological functions [63]. In addition to being color compounds and accessory pigments in the photosynthesis, they are also known as antioxidants and UV protecting molecules [64,65]. Former studies have shown that algae and higher plants increased the production of carotenoids in response to stresses such as UV light associated damage, nutrient depletion, pH and temperature [65,66,67,68]. Although no studies were found relating grazing stress to the production of carotenoids it is plausible that this may have a connection.

In conclusion, the present study reveals that grazing zooplankton have an effect on the temperate diatom *P. glacilias*, and that the effect can be seen in both the metabolic profile and the expressed bioactivity.

## 4. Materials and Methods

### 4.1. Mass Cultivation of Porosira Glacialis

Two nonaxenic batch cultures of *P. glacialis* were cultivated at an outdoor mass cultivation facility, in a 6000 L glass fiber vertical column open photobioreactor. As irradiance from natural light is scarce and the solar angle is low during winter in Northern Norway (69°13′0″ N, 18°5′10″ E), the cultures were illuminated using LED light (500–700 W) at photoperiod 24 h to provide consistent light conditions. Seawater used in the cultivations was from an inlet at 25 m depth filtrated through a series of filters; 5 µm particle filter (Azud, Murcia, Spain), 1 µm filter cartridge (Eaton, Dublin, Ireland) and a UV unit (450 mJ cm^−1^ at 12 m^3^ t^−1^) (ULTRAAQUA A/S, Aalborg, Demark). Silicate solution and inorganic nutrients (N, P, Mg, K, S and Fe) were added in order to allow the microalgae to grow at nutrient replete conditions. Daily measurements of nutrient concentrations (NO_3_^−^, SiO_2_ and PO_4_^3−^), temperature (°C) and pH were done to monitor cultivation conditions. In addition, biomass concentrations were inferred by cell counts [69] and chlorophyll concentration measurements (raw fluorescence). Daily visual examination was performed to assess culture health using an inverted microscope (Zeiss Axiovert A1, Carl Zeiss Microscopy GmbH, Jena, Germany). Diatom biomass was harvested after 5 days of growth using a continuous centrifuge (Evodos 10, Evodos B.V., Raamsdonksveer, The Netherlands), frozen immediately after harvest and kept at −80 °C until further use. After the first harvest the diatom culture was diluted and prepared for the zooplankton challenge experiment.

### 4.2. Zooplankton Collection

**Figure 8:**
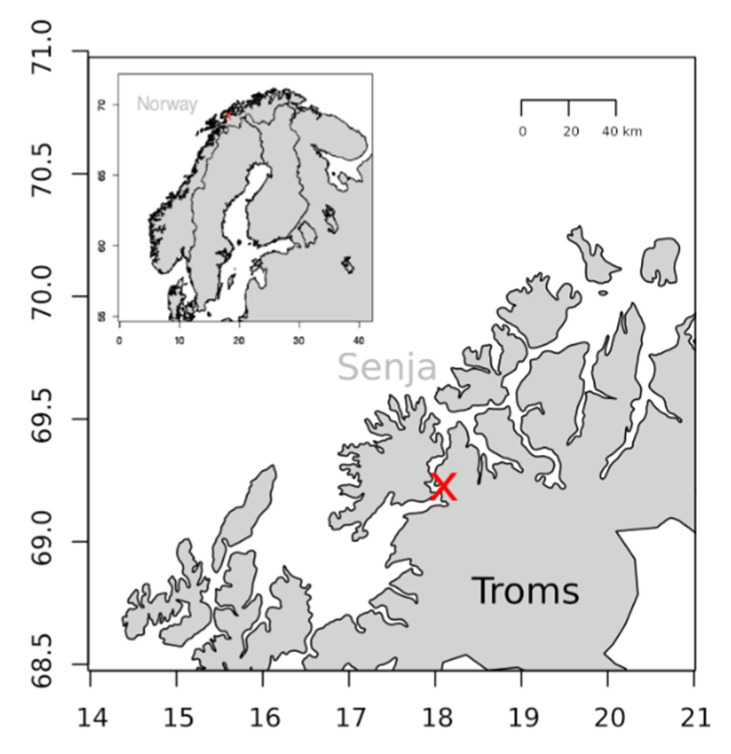
The map shows the area where the zooplankton samples for the cocultivation experiment were collected. The map was generated using the package “Maps” version 3.1.1. in R version 3.3.2.

Samples of zooplankton were collected near the shore in Finnfjordbotn in Northern Norway (Figure 8) using a WP2 plankton net (180 μm) with a detachable cod-end. The net was towed at ca. 1 knots towing speed just below the surface at the side of a Polarcircle 560 Work boat. Each WP-2 haul lasted for about 20–30 min before the net catch was emptied into sample flasks. The transfer was done quickly due to low air temperature (ca. −13 °C) and risk of sea water freezing. All net samples were pooled and stored at approximate seawater temperature at the sampling site, i.e., 2 °C in an insulated container onboard until use (2–4 h). Larger species, such as ctenophores were removed from the sample. Subsamples were preserved in 96% ethanol and stored for taxonomic analysis.

### 4.3. Cocultivaion

**Figure 9:**
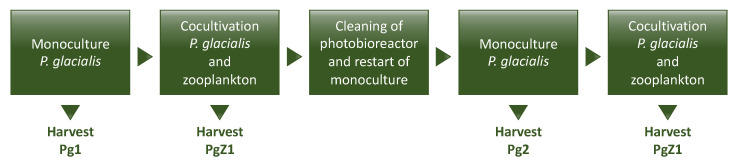
Overview of experimental workflow.

The collected zooplankton sample was transferred to the 6000 L open photobioreactor containing the *P. glacialis* monoculture. *P. glacialis* was cocultivated with the field zooplankton sample. The other cultivation parameters and daily measurements were the same as for the monoculture. As a survival control, flasks (2 L) with the coculture of *P. glacialis* and the zooplankton sample were kept in a cultivation incubator in the lab at 4 °C with a photoperiod of 14:10 (light:dark) to monitor if the zooplankton survived in the dense culture. Feeding/no feeding was monitored by inspecting zooplankton gut content after 2 days, using an inverted microscope at 100× magnification (Zeiss Axiovert A1, Carl Zeiss Microscopy GmbH, Germany). Figure 9 shows an overview of the cultivation and cocultivation pipeline. For each cocultivation new zooplankton bulk samples were collected from a nearby bay area. The harvest of the coculture was done using a continuous centrifuge (Evodos 10, Evodos B.V., The Netherlands), but the culture was filtered through a plankton net (180 µm) prior to the centrifugation to make sure that zooplankton were kept from entering the centrifuge. Harvested biomass was frozen immediately and kept at −80 °C until use.

### 4.4. Extraction and Flash-Fractionation

All diatom biomass for bioactivity testing was freeze-dried, ground into a fine powder using mortar and pestle, and extracted overnight using MilliQ-H_2_O. It was then centrifuged at 4600 rpm and 4 °C for 30 min, and the supernatant was kept. The pellet was resuspended in MQ-H_2_O and extracted a second time for 30–60 min, and centrifuged. The supernatant was frozen at −80 °C, then freeze-dried and ground into a fine powder before being frozen at −20 °C. The extracted pellet was freeze-dried before being extracted overnight using a 1:1 mixture (vol:vol) of methanol (Sigma-Aldrich, St. Louis, MO, USA) and dichloromethane (Merck, Darmstadt, Germany). The extract was filtered, and the pellet was extracted a second time. The combined organic extract was dried under reduced pressure using a rotavapor (Laborata 4002, Heidolph Instruments GmbH, Schwaback, Germany).

Aqueous and organic extracts were fractionated using a flash purification system (Biotage HPFC SP4, Biotage^®^, Uppsala, Sweden). Organic extracts were prepared by suspending 1.5 g of extract in Hexane (40 mL g^−1^) in a separation funnel. MeOH (90%) was added (30 mL × 2), the solution mixed carefully, and the lower liquid phase (MeOH) was transferred to a round flask prefilled with 2 g of Dianon HP-20SS resin (Supelco, Bellefonte PA, USA) and dried under reduced pressure. Aqueous extracts were prepared by mixing 2 × 0.75 g of dried extract with 4 mL 90% MeOH, 1.5 g Dianon HP-20SS resin and 1 mL MilliQ-H_2_O, before being dried at reduced pressure.

Columns for flash-fractionation were prepared by washing 6.5 g of resin with MeOH for 20 min. MeOH was then exchanged with MQ-H_2_O, and the material was transferred to a flash column (Biotage^®^ SNAP, Uppsala, Sweden). The column was equilibrated with 5 % MeOH, and the dried extract was loaded to the column. Flash fractionation of the sample was done in two steps; first with a gradient of 5–100% MeOH and MilliQ-H_2_O at a flow rate of 12 mL min^−1^ for 32 min, and then a MeOH:acetone (Merck, Germany) gradient ending at 100% acetone, flow rate 12 mL min^−1^ over 18 min. The eluent was collected into eight fractions and dried under reduced pressure. Fractions were dissolved in 100% DMSO to a concentration of 40 mg mL^−1^. Deep well screening plates were prepared by adding 25 μL (i.e.,1 mg dry material) of each fraction and stored at −20 °C until screening. Fractions were resuspended in 975 μL dH_2_O right before screening.

### 4.5. Biofilm Assay

All flash-fractionated samples were tested for inhibition of biofilm formation using the bacterial strain *Staphylococcus epidermidis* (ATCC-35984) which is known to form biofilm. Bacterial colonies were transformed from blood agar to a liquid Tryptic Soy Broth enrichment media (TBS, Merck, Germany) and incubated at 37 °C overnight, and then diluted 1:100 in TBS + 1% glucose. The assay was performed in 96-well microtiter plates at a concentration of 100 μg/mL, and all fractions were tested in triplicates. 50 μL of each fraction and 50 μL of bacterial suspension was added to each well. The nonbiofilm forming bacteria *Staphylococcus haemolyticus* was used as negative control, *S. epidermidis* + dH_2_O as a positive control and TBS (1% glucose) and dH_2_0 as blank. Plates were incubated at 37 °C overnight. The bacterial suspension was removed carefully, and the wells were washed using MQ-H_2_O. To fixate the biofilm, the plates were stored at 55 °C for an hour. Then the biofilm was stained using 70 μL of 0.1% crystal violet (Merck, Germany) for 5 min. The liquid was removed by repeated washing with MQ-H_2_0, dried at 55 °C, and resuspended using 70% EtOH. Absorbance was read in a VICTOR 1420 Multilabel Counter (Perkin Elmer, Waltham, MA, USA) at 600 nm.

### 4.6. Growth Inhibtion Assay

Antibacterial activity was investigated in a growth inhibition assay. A panel of five different bacterial strains were used, and both Gram negative and positive bacteria were included. Colonies of *Staphylococcus aureus* (ATCC 25923), *Escherichia coli* (ATCC 25922) and *Pseudomonas aeruginosa* (ATCC 27853) were transferred from blood agar plates an inoculated at 37 °C for 24 h in Mueller–Hinton growth media (MH, Becton Dickinson Company, Franklin Lakes, NJ, USA), and *Escherichia faecalis* (ATCC 29212) and *Streptococcus agalactiae* (ATCC 12386) in brain heart infusion media (BHI, Sigma-Aldrich, Germany). After incubation 2 mL of each suspension was transferred to 25 mL of fresh growth medium and incubated until log-phase, before being diluted to 1:1000 to adjust cell density. The assay was performed in 96-well titer plates at 100 μg mL^−1^. 50 μL of each fraction and 50 μL of bacterial suspension was added to each well. All fractions were tested in duplicates. Growth medium + dH_2_O was used as negative control, and gentamicin was used as positive control. The plates were incubated at 37 °C overnight and absorbance was measured in a VICTOR 1420 Multilabel Counter at 600 nm.

### 4.7. Cell Viability Assay

The flash fractions were tested against two cancer cell lines: human melanoma (A2058, LGC Standards ATCC-CRL-11147) and human colon carcinoma (HT29, LGC Standards ATCC HTB-38), and for comparison, against normal lung fibroblasts (MRC5, LGC Standards ATCC CCL-171). 2000 cells were seeded into each well of a 96-well micro titer plate (4000 cells/well for MRC5 cell line) in Roswell Park Memorial Institute 1640 cell medium (RPMI) with 10% fetal bovine serum added 10 mg mL gentamicin and incubated at 37 °C and 5% CO2 for 2 h. After incubation the medium was replaced with 50 μL per well and the cells were exposed to 100 μg mL^−1^ of flash fractions for 72 h. All fractions were tested in triplicate. Culture medium was used as a negative control, while 10% (*v/v*) DMSO (Sigma-Aldrich, Germany) was used as the positive control. At the end of incubation, 10 μL of CellTiter 96 Aqueous One Solution Reagent (Promega, Madison, WI, USA) was added and incubated for 1 h. Then the absorbance was read at 485 nm using a DTX 880 Multimode Detector. Results were calculated as % cell survival, and survival below 50% was counted as active.

### 4.8. UHPLC-ESI-HR-MS Analysis and Data Processing for Metabolomic Profile

The UPLC-HR-MS analysis was done using a Waters Acquity I-class UPLC system (Waters, Milford, MA, USA) coupled to a PDA Detector and a VION IMS-qTOF, using electrospray ionization (ESI) in positive mode. Wavelengths from 190–500 nanometers were detected. VION IMS-qTOF conditions for UPLC-HR-MS analysis; capillary voltage (0.80 kV), cone gas (50 L h^−1^), desolvation temperature (350 °C), desolvation gas (800 L h^−1^), source temperature (120 °C) and acquisition range was *m/z* 50–2000. Chromatographic separation was performed with a BEH C18 1.7 μm (2.1 × 100 mm) column (Waters, Milford, MA, USA) maintained at 40 °C. Data from the analysis of flash fractions were processed using UNIFI 1.9.4 software (Waters). Selected peaks were dereplicated using MarinLit, ChemSpider, and Dictionary of Natural products, as well as extensive literature searches. Statistical analysis of metabolomics data was done using EZinfo v.3.0.3.0 (Umetris ab) and Progenesis QI v.2.4 (Nonlinar Dynamics) software for analysis of LC-MS data. Scores plot and s-plot were made using EZinfo.

### 4.9. Statistics and Software

Statistical analysis of data was done by running chi-square tests in Excel for Mac 2020 version 16.36. Figures were made using Prism 8 for Mac (GraphPad Software Inc.) and in Rstudio version 1.2.1335 [70]. Map was made with Maps version 3.1.1. in R version 3.3.2. [71].

## 5. Conclusions

Investigation of the metabolic profiles from the monocultures and the cocultures revealed metabolomic differences indicating that stress from grazing affected, e.g., the production of carotenoids in *P. glacialis*. Cocultivation with zooplankton also induced the production of compounds with cytotoxic activity towards normal lung fibroblasts. Using cocultivation to enhance or trigger the production of bioactive compounds in bacteria is quite common and this method can also be used in microalgae biodiscovery. Future OSMAC studies on diatom and zooplankton cocultivation should include cultivation also using alternative species of diatoms and zooplankton, as well as different concentrations of grazers and cultivation at other times of the year. Full genome sequencing of diatoms has revealed that their large genomes most likely harbor “silent” gene clusters. It is therefore reasonable to believe that diatoms have a greater potential to produce secondary metabolites than what emerges in “static” laboratory conditions and cultivation in monoculture.

## Figures and Tables

**Table 1:** Species identification and composition (%) of both zooplankton batches. Details on zooplankton quantity can be found in Table S1.

Species	Quantity (%)	
	PgZ1	PgZ2
*Acartia longiremis*	10.0	-
*Calanus finmarchicus*	2.6	-
*Calanus nauplius*	-	2.3
*Centopages typicus*	1.3	2.3
*Metridia longa*	0.3	-
*Microcalanus sp.*	2.4	-
*Oithona sp.*	65.1	53.5
*Pseudocalanus acuspes/sp.*	16.4	39.5
*Temora longa*	0.2	-
*Calanoida, uid juvenile*	1.0	-
*Paraeuchaeta norwegica*	-	2.3
*Bryozoa/cyphonautes*	0.8	-

## Data Availability

Data sets will be made available at UiT Open Reseach Data.

## Supplementary Materials

The following are available online at https://www.mdpi.com/1660-3397/19/2/87/s1, Figure S1: Temperature and salinity profiles of zooplankton sampling sites; Figure S2: Daily temperature measurements; Figure S3: Daily pH measurements; Figure S4: Nitrate measurements; Figure S5: Silicate measurements; Figure S6: Phosphate measurements; Table S1: Zooplankton composition of bulk zooplankton samples; Table S2: Statistical analysis of bioactivity.

## References

[1] Demain A.L., Sanchez S. (2009). Microbial drug discovery: 80 years of progress. J. Antibiot..

[2] Romano S., Jackson S.A., Patry S., Dobson A.D. (2018). Extending the “one strain many compounds” (OSMAC) principle to marine microorganisms. Mar. Drugs.

[3] Bode H.B., Bethe B., Höfs R., Zeeck A.J.C. (2002). Big effects from small changes: Possible ways to explore nature’s chemical diversity. ChemBioChem.

[4] Uchoa P.K.S., Pimenta A.T.A., Braz-Filho R., Oliveira M.D.C.F.D., Saraiva N.N., Rodrigues B.S.F., Pfenning L.H., Abreu L.M., Wilke D.V., Florêncio K.G.D. (2017). New cytotoxic furan from the marine sediment-derived fungi Aspergillus niger. Nat. Prod. Res..

[5] Zhu F., Chen G., Chen X., Huang M., Wan X. (2011). Aspergicin, a new antibacterial alkaloid produced by mixed fermentation of two marine-derived mangrove epiphytic fungi. Chem. Nat. Compd..

[6] Okami Y., Okazaki T., Kitahara T., Umezawa H. (1976). Studies on marine microorganisms. V. A new antibiotic, aplasmomycin, produced by a streptomycete isolated from shallow sea mud. J. Antibiot..

[7] Pan R., Bai X., Chen J., Zhang H., Wang H. (2019). Exploring Structural Diversity of Microbe Secondary Metabolites Using OSMAC Strategy: A Literature Review. Front. Microbiol..

[8] Si Y., Tang M., Lin S., Chen G., Feng Q., Wang Y., Hua H., Bai J., Wang H., Pei Y.-H. (2018). Cytotoxic cytochalasans from *Aspergillus flavipes* PJ03-11 by OSMAC method. Tetrahedron Lett..

[9] Sung A.A., Gromek S.M., Balunas M.J. (2017). Upregulation and Identification of Antibiotic Activity of a Marine-Derived Streptomyces sp. via Co-Cultures with Human Pathogens. Mar. Drugs.

[10] Jensen P.R., Fenical W. (1996). Marine bacterial diversity as a resource for novel microbial products. J. Ind. Microbiol. Biotechnol..

[11] Mann D.G. (1999). The species concept in diatoms. Phycologia.

[12] Lauritano C., Andersen J.H., Hansen E., Albrigtsen M., Escalera L., Esposito F., Helland K., Hanssen K.Ø., Romano G., Ianora A. (2016). Bioactivity Screening of Microalgae for Antioxidant, Anti-Inflammatory, Anticancer, Anti-Diabetes, and Antibacterial Activities. Front. Mar. Sci..

[13] Ingebrigtsen R.A., Hansen E., Andersen J.H., Eilertsen H.C. (2016). Light and temperature effects on bioactivity in diatoms. Environ. Boil. Fishes.

[14] Degerlund M., Eilertsen H.C. (2009). Main Species Characteristics of Phytoplankton Spring Blooms in NE Atlantic and Arctic Waters (68–80° N). Chesap. Sci..

[15] Braarud T., Gaarder K.R., Nordli O. (1958). Seasonal changes in the phytoplankton at various points off the Norwegian West Coast:(Observations at the permanent oceanographic stations, 1945–1946). Rep. Nor. Fish. Mar. Investig..

[16] Wiborg K.F. (1954). Investigations on zooplankton in coastal and offshore waters of western and northwestern Norway—With special reference to the copepods. Rep. Nor. Fish. Mar. Investig..

[17] Cushing D. (1989). A difference in structure between ecosystems in strongly stratified waters and in those that are only weakly stratified. J. Plankton Res..

[18] Legendre L. (1990). The significance of microalgal blooms for fisheries and for the export of particulate organic carbon in oceans. J. Plankton Res..

[19] Caldwell G. (2009). The Influence of Bioactive Oxylipins from Marine Diatoms on Invertebrate Reproduction and Development. Mar. Drugs.

[20] Gudimova E., Eilertsen H.C., Jørgensen T.Ø., Hansen E. (2016). In vivo exposure to northern diatoms arrests sea urchin embryonic development. Toxicon.

[21] Harðardóttir S., Pančić M., Tammilehto A., Krock B., Møller E.F., Nielsen T.G., Lundholm N. (2015). Dangerous Relations in the Arctic Marine Food Web: Interactions between Toxin Producing *Pseudo-nitzschia* Diatoms and *Calanus* Copepodites. Mar. Drugs.

[22] Ianora A., Miralto A. (2009). Toxigenic effects of diatoms on grazers, phytoplankton and other microbes: A review. Ecotoxicology.

[23] Ianora A., Poulet S.A., Miralto A. (1995). A comparative study of the inhibitory effect of diatoms on the reproductive biology of the copepod *Temora stylifera*. Mar. Biol..

[24] Ianora A., Poulet S.A., Miralto A. (2003). The effects of diatoms on copepod reproduction: A review. Phycologia.

[25] Lauritano C., Borra M., Carotenuto Y., Biffali E., Miralto A., Procaccini G., Ianora A. (2011). First molecular evidence of diatom effects in the copepod *Calanus helgolandicus*. J. Exp. Mar. Biol. Ecol..

[26] Pohnert G. (2005). Diatom/Copepod Interactions in Plankton: The Indirect Chemical Defense of Unicellular Algae. ChemBioChem.

[27] Pohnert G., Lumineau O., Cueff A., Adolph S., Cordevant C., Lange M., Poulet S. (2002). Are volatile unsaturated aldehydes from diatoms the main line of chemical defence against copepods?. Mar. Ecol. Prog. Ser..

[28] Wichard T., Poulet S.A., Halsband-Lenk C., Albaina A., Harris R., Liu D., Pohnert G.J. (2005). Survey of the chemical defence potential of diatoms: Screening of fifty species for α, β, γ, δ-unsaturated aldehydes. J. Chem. Ecol..

[29] Ianora A., Poulet S.A. (1993). Egg viability in the copepod *Temora stylifera*. Limnol. Oceanogr..

[30] Miralto A., Barone G., Romano G., Poulet S.A., Ianora A., Russo G.L., Buttino I., Mazzarella G., Laabir M., Cabrini M. (1999). The insidious effect of diatoms on copepod reproduction. Nat. Cell Biol..

[31] Chaudron Y., Poulet S.A., Laabir M., Ianora A., Miralto A. (1996). Is hatching success of copepod eggs diatom density-dependent?. Mar. Ecol. Prog. Ser..

[32] Poulet S., Laabir M., Ianora A., Miralto A. (1995). Reproductive response of *Calanus helgolandicus*. I. Abnormal embryonic and naupliar development. Mar. Ecol. Prog. Ser..

[33] Pohnert G. (2000). Wound-Activated Chemical Defense in Unicellular Planktonic Algae. Angew. Chem. Int. Ed..

[34] Selander E., Kubanek J., Hamberg M., Andersson M.X., Cervin G., Pavia H. (2015). Predator lipids induce paralytic shellfish toxins in bloom-forming algae. Proc. Natl. Acad. Sci. USA.

[35] Selander E., Thor P., Toth G., Pavia H. (2006). Copepods induce paralytic shellfish toxin production in marine dinoflagellates. Proc. R. Soc. B Boil. Sci..

[36] Bergkvist J., Thor P., Jakobsen H.H., Wängberg S.Å., Selander E. (2012). Grazer-induced chain length plasticity reduces grazing risk in a marine diatom. Limnol. Oceanogr..

[37] Selander E., Berglund E.C., Engström P., Berggren F., Eklund J., Harðardóttir S., Lundholm N., Grebner W., Andersson M.X. (2019). Copepods drive large-scale trait-mediated effects in marine plankton. Sci. Adv..

[38] Lundholm N., Krock B., John U., Skov J., Cheng J., Pančić M., Wohlrab S., Rigby K., Nielsen T.G., Selander E. (2018). Induction of domoic acid production in diatoms—Types of grazers and diatoms are important. Harmful Algae.

[39] Krug D., Müller R. (2014). Secondary metabolomics: The impact of mass spectrometry-based approaches on the discovery and characterization of microbial natural products. Nat. Prod. Rep..

[40] Wishart D.S. (2016). Emerging applications of metabolomics in drug discovery and precision medicine. Nat. Rev. Drug Discov..

[41] Matsuno T., Ookubo M. (1981). A new carotenoid, halocynthiaxanthin from the sea squirt. Tetrahedron Lett..

[42] Liaaen-Jensen S., Renstrøm B., Ramdahl T., Hallenstvet M., Bergquist P. (1982). Carotenoids of Marine Sponges. Biochem. Syst. Ecol..

[43] Litchfield C., Liaaen-Jensen S. (1980). Carotenoids of the marine sponge *Microciona prolifera*. Comp. Biochem. Physiol. Part B Comp. Biochem..

[44] Mosadeghzad Z., Zuriati Z., Asmat A., Gires U., Wickneswari R., Pittayakhajonwut P., Farahani G. (2013). Chemical components and bioactivity of the marine-derived fungus *Paecilomyces* sp. collected from Tinggi Island, Malaysia. Chem. Nat. Compd..

[45] Huseby S., Degerlund M., Eriksen G.K., Ingebrigtsen R.A., Eilertsen H.C., Hansen E. (2013). Chemical Diversity as a Function of Temperature in Six Northern Diatom Species. Mar. Drugs.

[46] Søreide J.E., Falk-Petersen S., Hegseth E.N., Hop H., Carroll M.L., Hobson K.A., Blachowiak-Samolyk K. (2008). Seasonal feeding strategies of *Calanus* in the high-Arctic Svalbard region. Deep. Sea Res. Part II Top. Stud. Oceanogr..

[47] Barth-Jensen C., Koski M., Varpe Ø., Glad P., Wangensteen O.S., Præbel K., Svensen C. (2020). Temperature-dependent egg production and egg hatching rates of small egg-carrying and broadcast-spawning copepods *Oithona similis*, *Microsetella norvegica* and *Microcalanus pusillus*. J. Plankton Res..

[48] Castellani C., Irigoien X., Harris R.P., Holliday N.P. (2007). Regional and temporal variation of *Oithona* spp. biomass, stage structure and productivity in the Irminger Sea, North Atlantic. J. Plankton Res..

[49] Norrbin M.F. (1994). Seasonal patterns in gonad maturation, sex ratio and size in some small, high-latitude copepods: Implications for overwintering tactics. J. Plankton Res..

[50] Durbin A.G., Durbin E.G. (1981). Standing stock and estimated production rates of phytoplankton and zooplankton in Narragansett Bay, Rhode Island. Estuaries.

[51] Preisser E.L., Bolnick D.I., Benard M.F. (2005). Scared to Death? The Effects of Intimidation and Consumption in Predator–Prey Interactions. Ecology.

[52] Amin S.A., Parker M.S., Armbrust E.V. (2012). Interactions between Diatoms and Bacteria. Microbiol. Mol. Biol. Rev..

[53] Sansone C., Braca A., Ercolesi E., Romano G., Palumbo A., Casotti R., Francone M., Ianora A. (2014). Diatom-derived polyunsaturated aldehydes activate cell death in human cancer cell lines but not normal cells. PLoS ONE.

[54] Ingebrigtsen R.A., Hansen E., Andersen J.H., Eilertsen H.C. (2017). Field sampling marine plankton for biodiscovery. Sci. Rep..

[55] Cheng H.-H., Wang H.-K., Ito J., Bastow K.F., Tachibana Y., Nakanishi Y., Xu Z., Luo T.-Y., Lee K.-H. (2001). Cytotoxic Pheophorbide-Related Compounds from Clerodendrum calamitosum and C. cyrtophyllum. J. Nat. Prod..

[56] Andrade K.A.M., Lauritano C., Romano G., Ianora A. (2018). Marine Microalgae with Anti-Cancer Properties. Mar. Drugs.

[57] Peng J., Yuan J.-P., Wu C.-F., Wang J.-H. (2011). Fucoxanthin, a Marine Carotenoid Present in Brown Seaweeds and Diatoms: Metabolism and Bioactivities Relevant to Human Health. Mar. Drugs.

[58] Cha K.H., Koo S.Y., Lee D.-U. (2008). Antiproliferative Effects of Carotenoids Extracted from *Chlorella ellipsoidea* and *Chlorella vulgaris* on Human Colon Cancer Cells. J. Agric. Food Chem..

[59] Bjaerke O., Jonsson P.R., Alam A., Selander E. (2015). Is chain length in phytoplankton regulated to evade predation?. J. Plankton Res..

[60] Amato A., Sabatino V., Nylund G.M., Bergkvist J., Basu S., Andersson M.X., Sanges R., Godhe A., Kiørboe T., Selander E. (2018). Grazer-induced transcriptomic and metabolomic response of the chain-forming diatom *Skeletonema marinoi*. ISME J..

[61] Gieskes W.W.C., Engelkes M.M., Grakaay G.W. (1991). Degradation of diatom chlorophyll to colourless, non-fluorescing compounds during copepoo grazing. Aquat. Ecol..

[62] Shuman F.R., Lorenzen C.J. (1975). Quantitative degradation of chlorophyll by a marine herbivore1. Limnol. Oceanogr..

[63] Chuyen H.V., Eun J.-B. (2017). Marine carotenoids: Bioactivities and potential benefits to human health. Crit. Rev. Food Sci. Nutr..

[64] Bertrand M. (2010). Carotenoid biosynthesis in diatoms. Photosynth. Res..

[65] Krinsky N.I., Johnson E.J. (2005). Carotenoid actions and their relation to health and disease. Mol. Asp. Med..

[66] Rmiki N.-E., Schoefs B., Lemoine Y., Prakash A., Rao J. (1999). Carotenoids and Stress in Higher Plants and Algae. Pesticides in Agriculture and the Environment.

[67] Steinbrenner J., Linden H. (2001). Regulation of Two Carotenoid Biosynthesis Genes Coding for Phytoene Synthase and Carotenoid Hydroxylase during Stress-Induced Astaxanthin Formation in the Green Alga *Haematococcus pluvialis*. Plant Physiol..

[68] Vidhyavathi R., Venkatachalam L., Ravi S., Ravishankar G.A. (2008). Regulation of carotenoid biosynthetic genes expression and carotenoid accumulation in the green alga *Haematococcus pluvialis* under nutrient stress conditions. J. Exp. Bot..

[69] Utermöhl H. (1931). Neue Wege in der quantitativen Erfassung des Plankton. (Mit besonderer Berücksichtigung des Ultraplanktons). SIL Proc. 1922–2010.

[70] RStudio Team (2021). RStudio: Inegrated Development Environment for R. http://www.rstudio.

[71] Becker R., Wilks A., Brownrigg R. (2016). Maps: Draw Graphical Maps R Package Version 3.1.1. https://cran.r-project.org/package=maps.

