# Supplementary material for: Adding Zooplankton to the OSMAC Toolkit: Effect of Grazing Stress on the Metabolic Profile and Bioactivity of a Diatom
Source: Mar Drugs. 2021 Feb 3;19(2):87. doi: 10.3390/md19020087 (PMC7913365; doi:10.3390/md19020087)
Supplement: Supplementary file 1 [file marinedrugs-19-00087-s001.pdf]

Adding zooplankton to the OSMAC toolbox: Effect of grazing stress on the metabolic profile and bioactivity of a diatom.

Renate Døving Osvik [1,2], Richard Andre Ingebrigtsen [2], Fredrika Norrbin[3], Jeanette Hammer Andersen[1], Hans Christian Eilertsen[2] and Espen Holst Hansen[1]

[1] Marbio, The Norwegian College of Fishery Science, Faculty of Biosciences, Fisheries and Economics, UiT – The Arctic University of Norway, N-9037 Tromsø, Norway; renate.d.osvik@uit.no (R.D.O); jeanette.h.andersen@uit.no (J.H.A.); espen.hansen@uit.no (E.H.H.)

[2] Microalgae and microbiomes, The Norwegian College of Fishery Science, Faculty of Biosciences, Fisheries and Economics, UiT – The Arctic University of Norway, N-9037 Tromsø, Norway; Richard.a.ingebrigtsen@uit.no (R.A.I); hans.c.eilertsen@uit.no (H.C.E)

[3] Arctic marine system ecology, Department of Arctic and Marine Biology, Faculty of Biosciences, Fisheries and Economics, UiT – The Arctic University of Norway, N-9037 Tromsø, Norway; fredika.norrbin@uit.no

* Correspondence: renate.d.osvik@uit.no (R.D.O); Tel.: +47-776-49-265 (R.O.)

Supplemental Information Table of Contents

CTD data and zooplankton samples

Figure S1: Temperature and salinity profiles of zooplankton sampling sites

Table S1: Zooplankton composition of bulk zooplankton samples

Culture conditions

Figure S2: Daily temperature measurements

Figure S3: Daily pH measurements

Figure S4: Nitrate measurements

Figure S5: Silicate measurements

Figure S6: Phosphate measurements

Bioactivity data

Table S2: Statistical analysis of bioactivity data

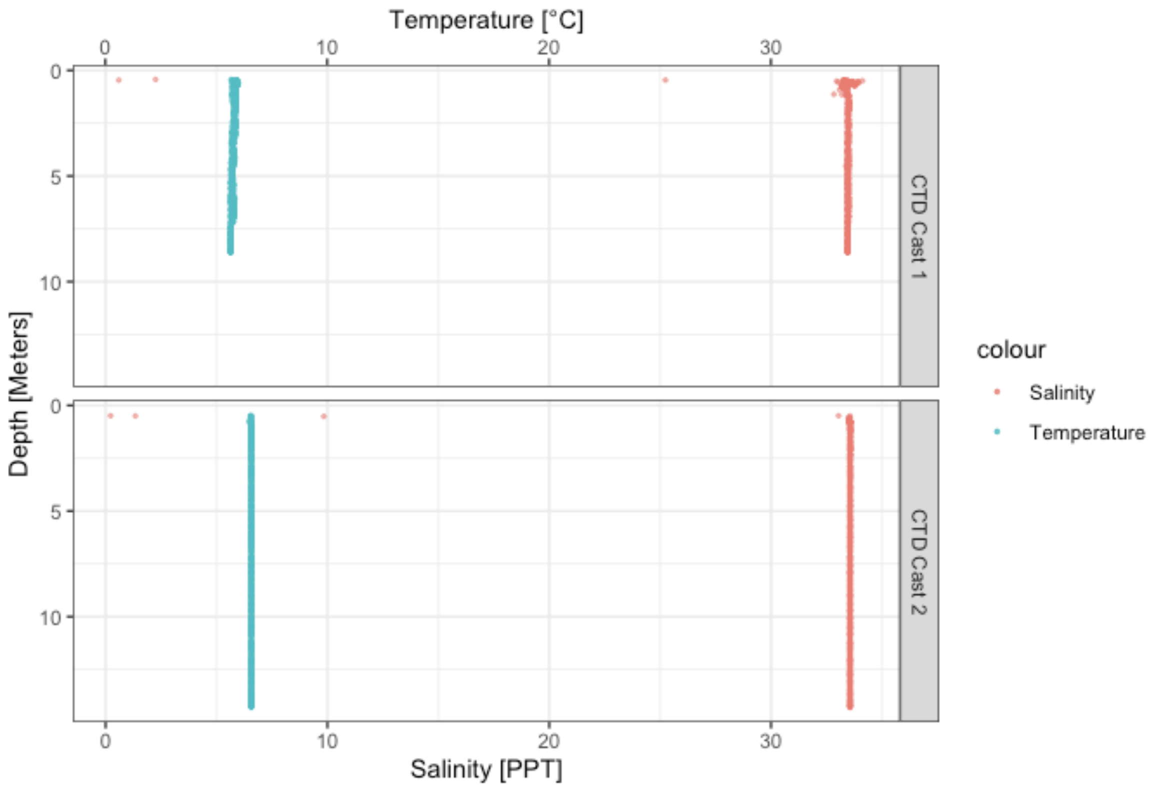

Supplementary Figure S1. Panel showing temperature and salinity profiles from the two sample sites where we collected zooplankton. The lower x-axis shows salinity as PPT with red dots and the upper x – axis shows temperature in degrees Celsius (°C) with blue dots, while the y-axis is depth in meters. CTD casts data were taken from surface to 50 cm above bottom and back to surface. Samples sites were similar, and the water column was well-mixed at both sites.

Supplementary Table S1: Zooplankton composition of bulk zooplankton samples. Zooplankton sub-samples were collected before zooplankton were added to culture of P. glacialis and stored in 96% EtOH.

**Species**	**Samples 1 (PgZ1)**			**Samples 2 (PgZ2)**		
	**Count**	%	**Comment**	**Count**	%	**Comment**
Acartia longiremis	62	9,98	mostly adult female			
Calanus finmarchicus	16	2,58				
Calanus nauplius				1	2,33	
Centopages typicus	8	1,29	copepodites	1	2,33	
Metridia longa	2	0,32				
Microcalanus sp	15	2,42	copepod/adults			
Oithina sp.	404	65,06	most stages	23	53,49	
Pseudocalanus acuspes/sp	102	16,43	copepod/adults	17	39,53	5 females
Temora longa	1	0,16	copepodite			
Calanoida, uid juvenile	6	0,97	mostly adult females	1	2,33	
Paraeuchaeta						
Bryozoa/cyphonautes	5	0,81				
Total	621	100		43	100	

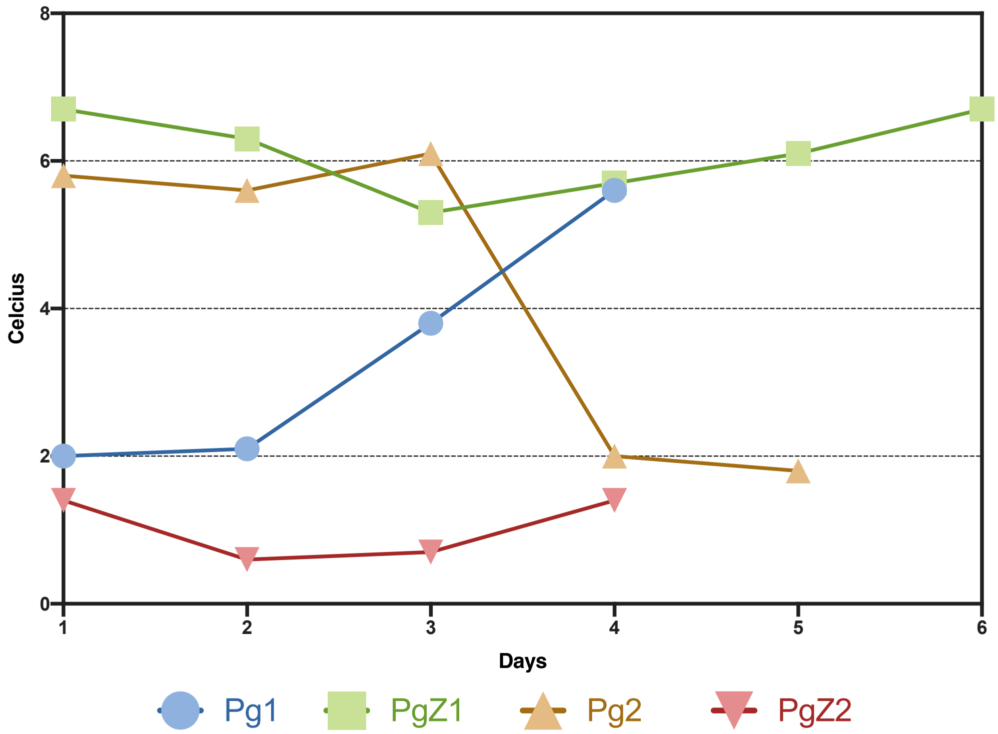

Supplementary Figure S2: Daily temperature measurements (in °C). The temperature was measured daily just beneath the surface of the culture.

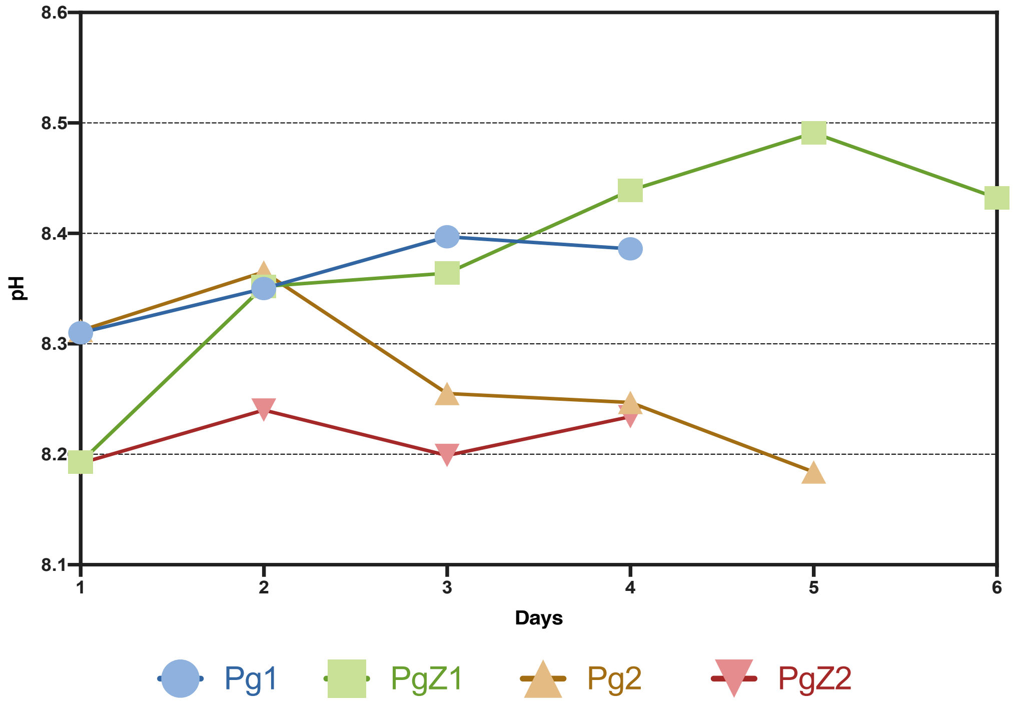

Supplementary Figure S3: Daily pH measurements. The pH was measured daily in all cultures using a pH meter just below the surface of the culture.

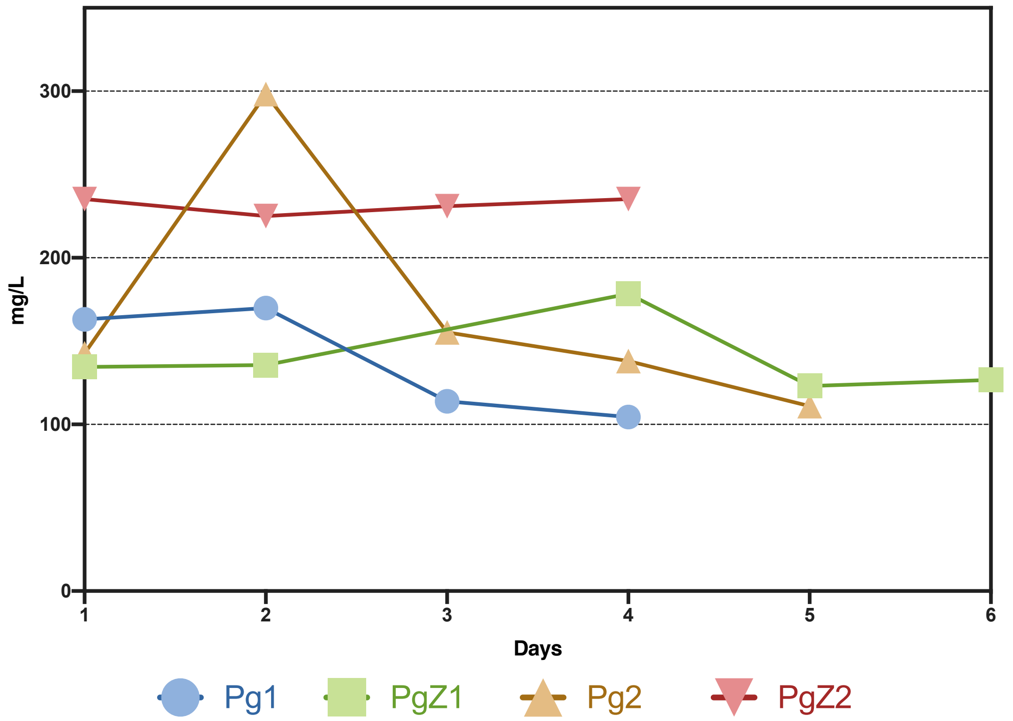

Supplementary Figure S4: Daily nitrate measurements of all cultures (in mg/L). Nitrate concentration was measured using Spectroquant® Nitrate test in seawater (Merck KGaA, 64271 Damstadt, Germany)

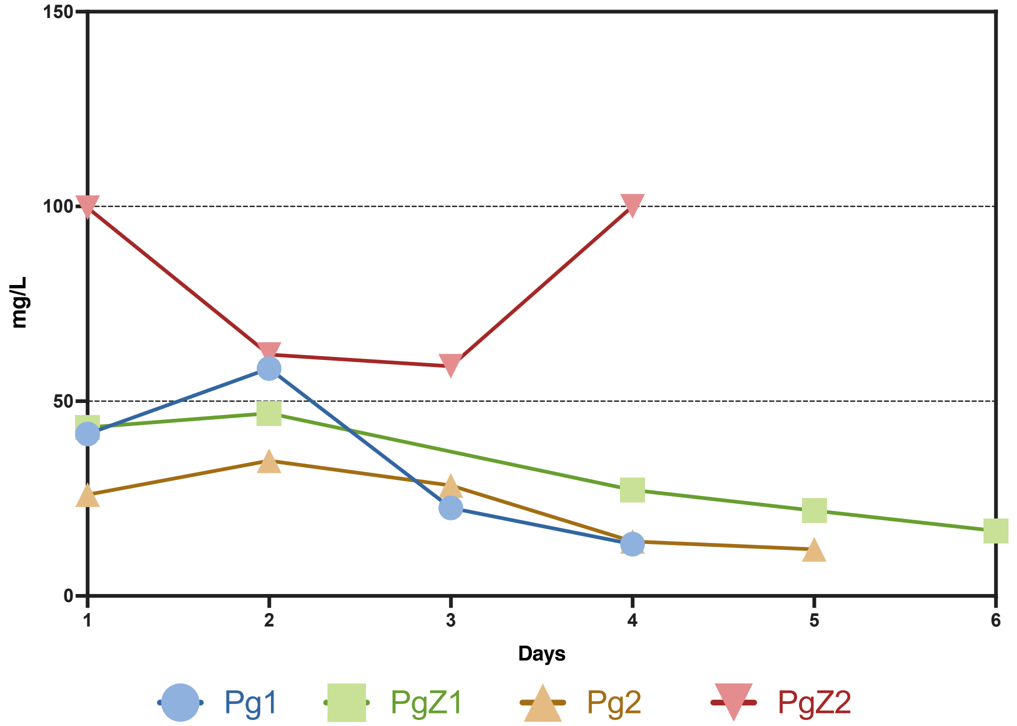

Supplementary Figure S5: Daily silicate measurements of all cultures (in mg/L). Silicate concentration was measured using Spectroquant® Silicate (Silicic Acid) Test (Merck KGaA, 64271 Damstadt, Germany)

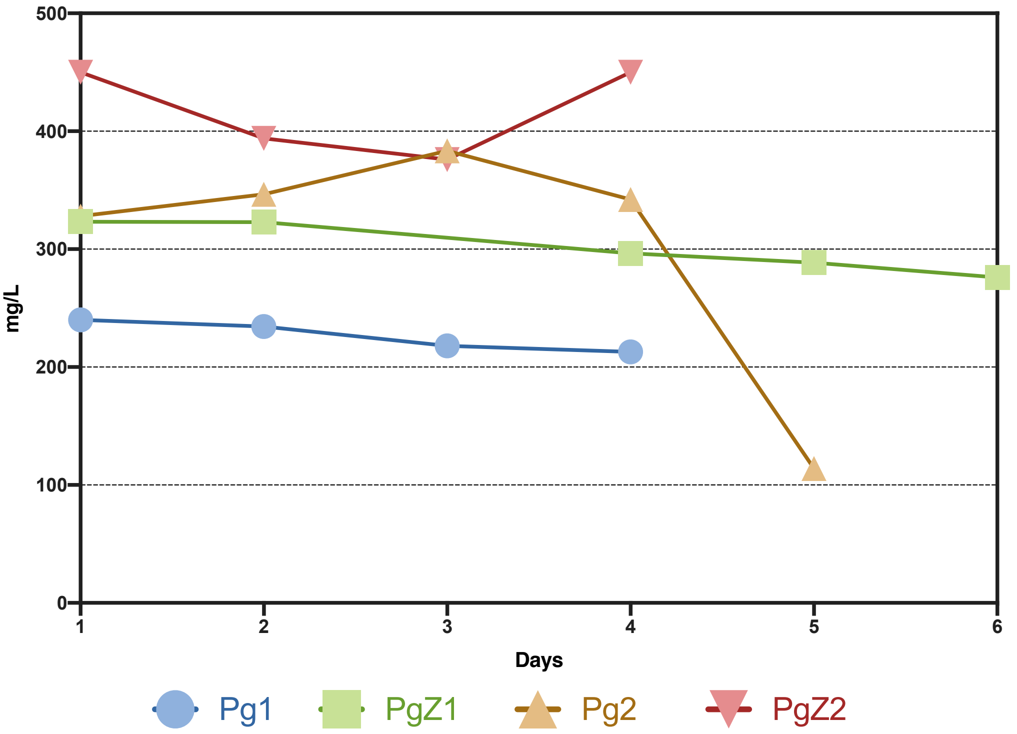

Supplementary Figure S6: Daily phosphate measurements of all cultures (in mg/L). Phosphate concentration was measured using Spectroquant® Phosphate Test (o-phosphate) (Merck KGaA, 64271 Damstadt, Germany)

Supplementary Table S2. Chi-square table showing p-values of the total number of active Flash - fractions versus inactive flash fractions between the different treatments.

Sample Content	**Pg1**	**Pg2**	**PgZ1**	**PgZ2**
**Pg1**	-	0.426	0.426	0.273
**Pg2**	0.426	-	1.000	0.426
**PgZ1**	0.426	0.759	-	0.759
**PgZ2**	0.273	0.426	0.759	-
